# Pituitary carcinoma - case series and review of the literature

**DOI:** 10.3389/fendo.2022.968692

**Published:** 2022-09-08

**Authors:** Stephanie Du Four, Jorn Van Der Veken, Johnny Duerinck, Elle Vermeulen, Corina E. Andreescu, Michael Bruneau, Bart Neyns, Van Velthoven, Brigitte Velkeniers

**Affiliations:** ^1^ Department of Neurosurgery, Universitair Ziekenhuis Brussel (UZ Brussel), Vrije Universiteit Brussel (VUB), Brussels, Belgium; ^2^ Department of Neurosurgery, AZ Delta, Roeselare, Belgium; ^3^ Department of Neurosurgery, Flinders Medical Centre, Adelaide, SA, Australia; ^4^ Department of Endocrinology, Universitair Ziekenhuis Brussel (UZ Brussel), Vrije Universiteit Brussel (VUB), Brussels, Belgium; ^5^ Department of Medical Oncology, Universitair Ziekenhuis Brussel (UZ Brussel), Vrije Universiteit Brussel (VUB), Brussels, Belgium; ^6^ Department of Internal Medicine, Universitair Ziekenhuis Brussel (UZ Brussel), Vrije Universiteit Brussel (VUB), Brussels, Belgium

**Keywords:** pituitary adenoma, surgery, immunotherapy, radiotherapy, PitNET, pituitary carcinoma, temozolomide

## Abstract

Although pituitary adenomas (PAs) account for 15% of intracranial tumors, pituitary carcinomas (PCs) are a rare entity. Most commonly, PCs evolve from aggressive PAs invading the surrounding structures and eventually leading to metastatic lesions. Due to the low incidence, the diagnosis and treatment remains challenging. We report a case series of five patients with pituitary carcinoma (PC) treated in our center. At first diagnosis 3 patients had an ACTH-producing adenoma, 1 a prolactinoma and 1 a double secreting adenoma (GH and prolactin). The mean time interval from initial diagnosis to diagnosis of PC was 10.7 years (range 5-20 years). All patients underwent multiple surgical resections and radiotherapy. Four patients were treated with temozolomide for metastatic disease. One patient with concomitant radiochemotherapy for local recurrence. Temozolomide led to a stable disease in 2 patients. One patient had a progressive disease after 9 cycles of temozolomide. In absence of standard treatment, immunotherapy was initiated, resulting in a stable disease.

We report five cases of PCs. Three patients obtained a stable disease after tailored multidisciplinary treatment. Additionally, one patient was treated with immunotherapy, opening a new treatment option in PCs. Overall, PCs are rare intracranial neoplasms occurring several years after the initial diagnosis of aggressive PAs. Currently, the absence of predictive factors for an aggressive clinical course, provokes a challenging management.

## Introduction

Pituitary adenomas are a commonly encountered pathology, with an overall estimated incidence of 16.7% (1).

Most often, pituitary adenomas (PAs) are slow growing tumors that do not invade into the surrounding tissues. However, 20 to 25% of PAs invade and infiltrate the surrounding structures including bone, cavernous sinuses and sphenoid sinus (2).

In the 2004 WHO classification three types of PAs were described: typical adenoma with low Ki-67 proliferative index, atypical adenoma characterized by Ki-67>3% and overexpression of p53, and carcinoma which demonstrate metastatic spread by craniospinal or systemic metastases (3). However, in the 2017 WHO classification the term atypical adenoma was abandoned due to the low predictive value of invasive behavior, based on the mitotic index and p53 (4, 5).

Pituitary carcinomas (PC) are very rare clinical entities with an incidence of 0.1 to 0.2% of pituitary tumors (6). The majority of PCs originate from functional PAs, most commonly from prolactin-secreting, followed by ACTH-secreting adenomas. Fifteen to twenty percent of PCs are non-functional, which comprises gonadotroph, silent corticotroph and rarely null cell carcinomas. The clinical presentation of patients with PCs is highly variable and depends on the functioning or non-functioning state, as well as the location and size of the metastatic lesions (7).

In the absence of prognostic criteria or pathological markers that reliably predict the behavior of PAs and due to the unpredictable clinical course, early identification of PCs is challenging but important to reduce morbidity and mortality in these patients (8, 9).

Currently, there are no prognostic criteria or pathological markers that reliably predict the behavior of PAs. The difficulty of early recognition of aggressive PAs, in combination with the absence of prognostic tools and low incidence of PC makes it difficult to adequately treat patients with PC. The aim of this study is to give an overview of the clinical evolution of different PAs that evolved over several years into PCs and set our clinical practice against the recently published data.

## Methods

Informed consent has been obtained from the patients still in follow-up for publication of the case reports and accompanying images. Since it concerns a case study and patients provided informed consent for publication an approval from the institution’s ethical commission is not required.

## Results

### Case 1

In 1994, a 34-year-old man developed a bitemporal hemianopsia due to a voluminous, cystic pituitary tumor. Biochemically the patient had no clinical signs or symptoms of Cushing disease and the ACTH and cortisol tests were within the normal range (ACTH 83pg/ml; cortisol 2,17μg/100ml). A transcranial resection was performed with recuperation of the visual field defect and preservation of the pituitary function.

Histological analysis showed an ACTH-secreting adenoma, with a Ki-67 less than 3%. As the postoperative MRI showed a millimetric remnant, the patient was treated with adjuvant radiotherapy.

In 2002, he suddenly developed a left fourth-nerve palsy due to compression of the cavernous sinus by a multilobulated cyst. A transsphenoidal endoscopic drainage of the cyst was performed, which resulted in a complete recovery of the fourth-nerve palsy but induced a panhypopituitarism.

In 2014, he developed an asymptomatic retroclival extra-axial mass encasing both vertebral arteries and compressing the medulla oblongata. In absence of other malignancies this lesion was most likely a metastasis. Because of the challenging location, the absence of clinical symptoms or biochemical abnormalities the decision for watchful waiting was made.

In 2015, this lesion caused a mass-effect on the medulla oblongata (**Figure 3B**) and a second lesion in the left cerebellar tonsil was diagnosed. Therefore, a surgical resection of the two lesions was performed. The histopathological analysis confirmed the metastasis of an ACTH-expressing adenoma. Neither obvious nuclear atypia nor mitotic activity was observed. The Ki-67 was less than 3% and p53 was negative.

A follow-up cerebral MRI 6 months postoperatively showed multiple new tumoral lesions at different intracranial locations. Due to the disseminated disease a treatment with temozolomide was initiated. After 12 cycles there was a slight decrease in size of the cerebellar and supratentorial lesions, the other lesions remained stable. Treatment was halted and at last follow-up in September 2021 there was no further clinical, biochemical or radiological progression.

### Case 2

In 2008, a 28-year-old male patient was diagnosed with a macroadenoma invading the right cavernous sinus and compressing the optic chiasm causing visual loss (**Figure 2A**). Biochemically there was an elevated IGF-1 (1167µg/l) and a discrete hyperprolactinaemia (25,19 ng/mL). The visual loss in absence of relevant hyperprolactinemia, indicated a staged surgical resection (transsphenoidal followed by transcranial resection).

The immunohistochemistry staining showed expression of growth hormone (GH) and prolactin (PRL) (Ki-67>10% and p53 20%).

Postoperatively, the increased IGF-1 (1053µg/L) persisted, however there was a loss of the corticotrophic, gonadotrophic and thyrotrophic function. Apart from hormonal substitution, a treatment with lanreotide and cabergoline was initiated. In the absence of a biochemical response, pegvisomant was added, without normalization of IGF1-levels.

Poor biochemical response and increasing neurological symptoms due to compression of the optic chiasm and invasion in the cavernous sinus, led to a third surgical resection. Postoperatively, a partial recovery of the vision was achieved, lanreotide, cabergoline and pegvisomant treatment was continued, followed by radiotherapy. After 6 weeks, a reduction of tumor volume and mass effect were observed. A normalization of IGF-1 and prolactin was reached at 6 months.

In 2012, an epileptic seizure and an isolated IGF-1 increase, led to the diagnosis of a left frontobasal lesion (**Figure 3A**). A surgical resection confirmed a metastatic lesion of a PRL and GH-secreting adenoma with high mitotic activity (5-6 mitoses per 10 HPF, p53 20%, Ki-67>3%).

In 2013, an asymptomatic left frontal metastasis was diagnosed on a cerebral MRI. A treatment with temozolomide was initiated (5 per 28 days, 150mg/m2) but due to persisting side-effects it had to be interrupted after three cycles. Subsequently, radiotherapy was performed (30 fractions of 1.8Gy) with complete resolution of the lesion and normalization of IGF-1.

In 2017, a left temporal metastasis was diagnosed due to recurrent epileptic seizures and increasing IGF-1. A surgical resection was performed with histological confirmation of a metastasis of a PC (p53 positive, Ki-67 25-35%, GH and PRL positive).

In 2019 there was an increase of PRL and IGF-1 due to a metastatic lesion in the petrous bone. A treatment with cabergoline and lanreotide was initiated resulting in a clinical and biochemical regression (**Figure 2A**: hormonal changes according to treatment).

In January 2020, an increasing IGF-1 and PRL led to the diagnosis of a new metastatic lesion in the clivus and sphenoid bone. Despite increasing doses of cabergoline and lanreotide there was a persistent increased IGF-1 and PRL. Thereupon fractionated radiotherapy was initiated in April 2021. At latest follow-up in July 2022 the patient had a normalization of the IGF-1 levels and a stabilization of the PRL levels.

### Case 3

In 1999, a 41-year-old patient was diagnosed with a macroprolactinoma causing visual field defects. A treatment with bromocriptine resulted in recovery of the visual field defects and biochemical normalization.

In 2008, the dosage of bromocriptine was increased for increasing PRL levels.

In 2011, the treatment with bromocriptine was interrupted and cabergoline was initiated for a persistent increase of PRL, however without success.

In 2012, he deteriorated clinically developing diplopia due to invasion of the cavernous sinus. Sequentially, a gross total resection was performed resulting in normalization of PRL levels and panhypopituitarism. Six months later, a quinagolide treatment was initiated for a recurrence with an increased PRL without radiological progression. Nonetheless, a persistent hyperprolactemia led to the initiation of fractionated radiotherapy (50Gy, 25 fractions of 2Gy) resulting in an involution of the remaining adenoma and a significant decrease of prolactin levels (422,48µg/L to 219,6µg/L).

In 2014, he had a biochemical and radiological recurrence. Besides a local recurrence, he also developed a retroclival mass at the level of the medulla oblongata, suggestive for a drop metastasis (**Figure 3C**). Temozolomide treatment was initiated and continued for 12 cycles resulting in a normalization of PRL levels and a slight tumor volume decrease.

In 2017, one year after the last dose of temozolomide, there was a progressive disease with increasing PRL levels. Temozolomide was restarted, but without success. Therefore, a transcranial surgical resection was performed. However, a total resection could not be obtained due to adherence to the optic chiasm and invasion of the cavernous sinus. Histological analysis confirmed a prolactinoma with a very high Ki-67 (>50%) and slightly increased p53 (5%).

In 2018, the patient had increasing visual field defects due to an increasing volume of the remnant adenoma. In absence of other treatment options, a contralateral transcranial resection was performed, and a gross total resection was achieved. The histological analysis confirmed a prolactinoma, however with a higher p53 expression (50%). The patient died in unclear circumstances in June 2018.

### Case 4

In 2014, a 58-year-old man, was diagnosed with Cushing’s disease due to a macroadenoma. A transsphenoidal resection confirmed the diagnosis of an ACTH-producing adenoma (Ki-67 <5% and p53 negative) and resulted in a hypocortisolemia necessitating a hydrocortisone treatment.

In 2015, increasing ACTH and cortisol led to the diagnosis of a recurrent macroadenoma.

Since he was under dual platelet therapy and anticoagulation therapy in the context of recent coronary artery stenting, a treatment with pasireotide was initiated awaiting surgery.

After 3 months, ketoconazole was added for persistently increased cortisol values, with obvious Cushing symptoms.

In 2016, a second transsphenoidal resection led to an initial decrease in cortisol levels. However, a bilateral adrenalectomy was performed 4 months after the surgery for a hypercortisolemia resistant to ketoconazole (**Figure 2B**: hormonal changes according to treatment).

In 2018, a recurrent invasive macroadenoma was detected on a routine cerebral MRI. A third transsphenoidal surgery was performed leaving tumor remnant in the cavernous sinus. There was a distinct change in histopathological analysis with the presence of a Ki-67 and p53 of more than 50%.

Two months after the surgery the patient developed a complete right sided visual loss due to compression of the right optic nerve. Concomitant fractionated radiotherapy and chemotherapy was started in combination with a high-dose corticosteroid treatment. Due to a renal insufficiency the treatment with temozolomide was interrupted. This treatment resulted in a volume reduction of the adenoma with a partial recovery of this vision.

In 2020, a routine check-up for osteoporosis diagnosed the presence of multiple bone metastases. An FDG-PET-CT scan and a biopsy of a skeletal metastasis confirmed the multiple metastases (**Figure 3E**). Given the frail general condition of the patient, it was decided, in agreement with the patient and his family, not to initiate any further treatment.

### Case 5

Our last case concerns a 33-year-old male patient with a Cushing’s disease and a bitemporal hemianopsia treated with surgical resection (transsphenoidal and transcranial resection) follow by a treatment with gamma knife radiotherapy and ketoconazole. Despite the patient developed cerebellar and drop metastases at the cervical spine (**Figure 3D**). Since surgical resection was considered unsafe, a treatment with temozolomide was initiated. After 9 cycles of temozolomide the patient had a progressive disease. A treatment with ipilimumab and nivolumab was initiated. The patient received four cycles of ipilimumab (3 mg/kg) and nivolumab (1 mg/kg) every 3 weeks, for 4 cycles in a compassionate use setting. The ACTH levels declined and the patient is currently still being treated with a maintenance of nivolumab every 4 weeks. This case was described and has been published (10).

## Discussion

We describe five cases of PC. In most cases, disease control was obtained after multimodal consisting of surgery, hormonal treatment, radiotherapy and chemotherapy, and immunotherapy (**Tables 1**, **2**, overview of local and systemic treatments). However, in two patients these treatments could not control the disease. Early diagnosis of aggressive PAs through predictive parameters of possible evolution into carcinoma are eagerly wanted in order to propose adequate treatment.

**Table 1:** Overview of the different local treatments including, surgery, radiotherapy and adrenalectomy.

	Case 1	Case 2	Case 3	Case 4	Case 5
**Sequence of local treatments**	transcranial surgery (1994)	transsphenoidal surgery (2008)	transsphenoidal surgery (2012)	transsphenoidal surgery (2014)	transsphenoidal surgery (2011)
	Radiotherapy (1994)	transcranial surgery (2009)	radiotherapy (2013)	transsphenoidal surgery (2016)	transcranial surgery (2012)
	transsphenoidal surgery (2002)	transsphenoidal surgery (2009)	transcranial surgery (2017)	bilateral adrenalectomy (2016)	radiotherapy (2013)
	resection of metastatic lesions (2015)	radiotherapy (2009)	transcranial surgery (2018)	transsphenoidal surgery (2018)	radiotherapy
		transcranial surgery (2012)		radiotherapy (2018)	bilateral adrenalectomy
		transcranial surgery (2017)			

**Table 2:** Overview of systemic treatment per case.

	Case 1	Case 2	Case 3	Case 4	Case 5
**Sequence of systemic treatments**	Temozolomide (2015)	Lanreotide (2008)	Bromocriptine (1999)	Pasireotide (2016)	Ketoconazole (2015)
		Pegvisomant + lanreotide (2009)	Cabergoline (2008)	Ketoconazole (2016)	Temozolomide (2018)
		Temozolomide (2013)	Quinagolide (2012)	Temozolomide (2018)	Ipilimumab + Nivolumab (2019)
		Cabergoline + lanreotide (2019)	Temozolomide (2013)		Nivolumab (2019)
			Temozolomide (2014)		

### Assessment of aggressiveness

The initial diagnosis of PAs is always confirmed with a full endocrinological work up and a cerebral MRI. A more detailed MRI of the sella turcica can show the presence of invasion of the surrounding structures. Some authors suggest this is indicative for an aggressive behavior (11).

The extensively used Knosp classification, which determines the extent of cavernous sinus invasion as compared to the internal carotid artery, helps in predicting the degree of resection, and hence the risk for recurrence. Based on this classification only grade 3 or grade 4 lesions are considered truly invasive (12, 13). The radiological classification guides the surgical management, and indication for adjuvant radiotherapy, but has inherent limitations. However, preoperative imaging is not fully reliable as invasion of the sellar floor or dura can be observed intraoperatively while it remained undetected on preoperative imaging (2, 11).

Recently, a clinicopathological classification combined radiological findings with immunocytological features (immunosubtype, Ki-67 index, mitotic count, and p53 positivity). The PAs were divided into 5 grades: grade 1a: non-invasive; grade 1b: non-invasive and proliferative; grade 2a: invasive; grade 2b: invasive and proliferative; grade 3: metastatic. At 8-years of follow-up they found that patients with grade 2b PAs at diagnosis, had a 12-to 25-fold probability of having tumor recurrence or progression as compared to those with grade 1a PAs. Moreover 6 of the 8 patients who developed a PC had a grade 2b PA at diagnosis (14).

**Figure 1:**
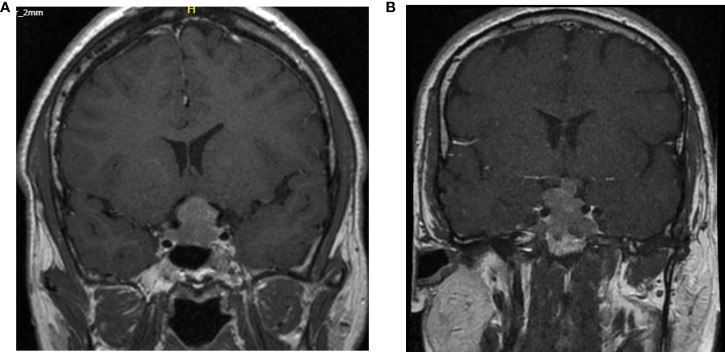
Cerebral MRI T1 weighted images with gadolinium at diagnosis of case 2 and case 5. **(A)** Knosp grade 4 classification of the second patient. **(B)** Knosp grade 3B classification of the 5^th^ patient.

In our case series 3 of the 5 patients were grade 4 in the Knosp classification at diagnosis (**Figure 1**).

Unfortunately, the Ki-67, mitotic count and p53 positivity was not systematically determined at the time of diagnosis. The second case can be classified as a grade 2b in the Trouillas classification. According to this classification and new guidelines a more aggressive treatment with an adjuvant radiotherapy after the first surgery would have been indicated.

A more unpredictable clinical course was present in the fifth patient. At initial diagnosis, there was a grade 1 Knosp classification and grade 1a according to Trouillas. Moreover, at recurrence the proliferative markers were also negative. However, a restaging of the 3^rd^ recurrence classified it as grade 4 (Knosp) and grade 2b (Trouillas) indicating a transformation towards an aggressive adenoma.

### Potential predictors of aggressiveness

In the European Society of Endocrinology (ESE) guidelines histopathological analysis with immunodetection of pituitary hormones and Ki-67 proliferative index are recommended. In case the Ki-67 is >3% an evaluation of the p53 immunodetection and mitotic count is indicated (15).

Additionally, the 2017 WHO classification encourages the use of transcription factors to detect a plurihormonal Pit-1-positive adenoma (a newly introduced high-risk PA) (5, 16, 17). This is emphasized in the new 2022 WHO classification were IHC plays an important role in the classification. In this classification the mammosomatotroph tumors are reported as a separate entity. Although we don’t have IHC confirmation, patient 2 could be considered as a mammasomatotroph adenoma (GH and PRL secreting adenoma) (18).

However, the use of p53 immunodetection and the mitotic count determination as prognostic tools are controversial, as illustrated in the present case series (17, 19–21).

The 2004 WHO classification established a 3% or higher cut off for Ki-67 labelling. This cut off was based on measurements in a single laboratory, without confirming their reproducibility (22). Several, but not all studies have shown an association between Ki-67 and invasiveness. These conflicting results are probably the result of different immunohistochemically techniques detecting Ki-67 (23). p53 is encoded by the tumor suppressor TP53 gene, which is almost never mutated in PAs. However, some studies found a significant increased p53 expression in recurrent tumors (24). Similar to Ki-67 the quantification of p53 varies by lab, however a common definition of >10 strongly positive nuclei per 10HPF was defined.

A recent study validated the clinicopathological classification of Trouillas et al. and thereby demonstrated the prognostic value of p53 (14, 25). When applying a Ki-67>3% and a positive p53 staining a strong prognostic value was reflected in a recent ESE survey where at least one pathology marker was available for 34 carcinomas: Ki-67 ≥3% was the most frequent positive marker in 85%, p53 positivity in 78% and a mitotic count in 90% (15).

In our patients the p53 and Ki-67 were determined in only 2 cases at initial diagnosis (**Table 3**). In the second patient they were already positive at initial diagnosis. When determined at recurrence, both markers were positive on the latest histological analysis in 3 cases.

**Table 3:** Overview of the evolution of p53 and Ki-67 in the different patients.

		p53	Ki-67
	**1994**	not determined	<3%
	**2002**	not determined	not determined
	**2015**	negative	<3%
	**2008**	not determined	>10% (positive)
	**2009**	not determined	not determined
	**2012**	20%	30%
	**2017**	20%	25-35%
	**2012**	not determined	not determined
	**2017**	5%	>50%
	**2018**	>50%	>50%
	**2014**	positive (light)	<5%
	**2016**	negative	<1%
	**2018**	positive (>50%)	>50%
	**2011**	not determined	not determined
	**2012**	not determined	not determined

### Genetic mutations

In three of the five patients (case 2, 3 and 4) a comparative genomic hybridization (CGH) genetic analysis of the tumor tissue was performed. All patients had chromosomal abnormalities that are related to a more invasive and aggressive tumor behavior. Allelic deletions at 4 different loci have been previously described (26).

In the second patient, an allelic deletion was present in two of these four loci: deletion on chr10q26 and chr13q12-14. This patient was also screened for the presence of *MEN-1 (multiple endocrine neoplasia type 1)* or *AIP* (aryl hydrocarbon receptor-interacting protein) mutations. However, the sequencing of both genes showed no abnormalities.

In the third case, deletion of chr1q and chr11q was combined with a gain of chr1q. Since it concerns a prolactinoma, these results correspond with the recent observations where the combination of a deletion of chr11q and gain of chr1q was found in aggressive prolactinoma (27).

In the fourth case, a gain of chr1q was found. That is also associated with a more aggressive evolution (28).

So, besides multiple chromosomal alterations all three patients have abnormalities that have been associated with aggressive evolution and malignancy in PAs.

### Pituitary carcinoma

When aggressive PAs develop metastases and become PCs the clinical symptoms are often site-related, with variable biochemical findings. The majority of PCs have endocrine activity and aggressive transformation most often occurs in ACTH and PRL-secreting PAs. The reported latency period for ACTH-secreting and PRL-secreting PCs was respectively 9.5 and 4.7years (16). This is similar to our patients in which the mean latency to malignant transformation was 10.7 years (resp. 20, 3, 1, 7 and 6years respectively) without a difference between the ACTH- and PRL-secreting PCs (resp. 10.5 and 11years). This is longer than reported in a case series by the university of MD Anderson Texas. They report 17 cases with a median time to PC conversion of 6 years. Similar to our cases the majority of PCs were hormone-secreting (29).

**Figure 2:**
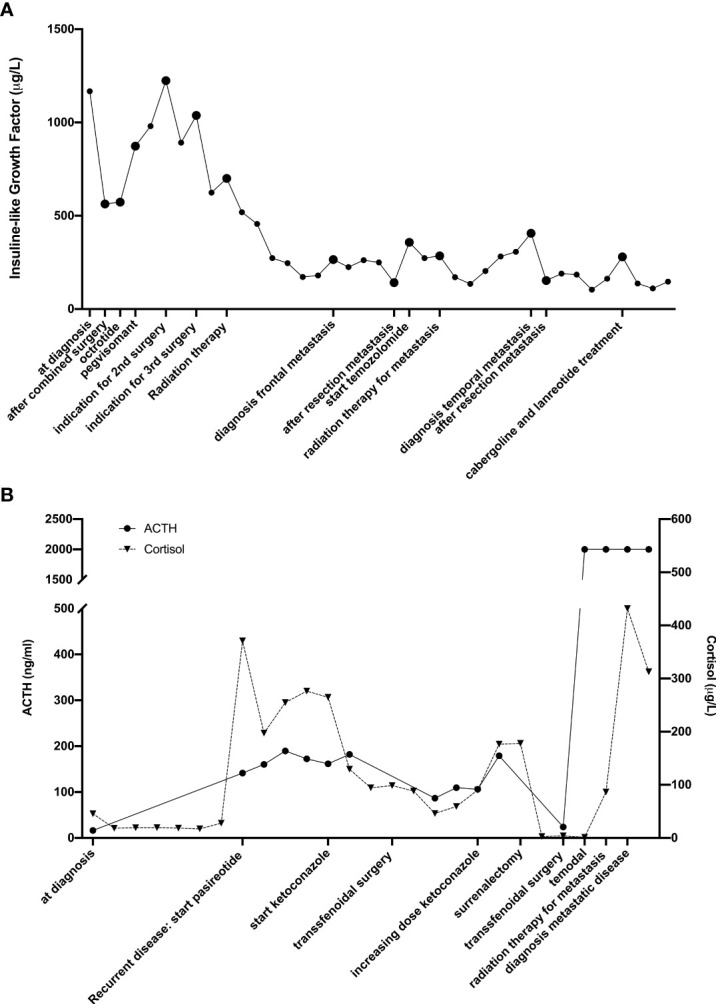
Timeline of hormonal changes in relation to the different treatments of the second **(A)** and fourth **(B)** patient.

The diagnosis of metastases is most often preceded by the development of new symptoms or hormonal changes. However, in some cases the metastases are asymptomatic and discovered accidently on surveillance imaging or post mortem (30). This was the case in 2 of the 5 patients. In **Figure 2**, we show the time evolution of the hormonal changes (respectively IGF-1 and ACTH) measurements in relation to the treatment and the development of metastases are shown for the second and fifth case.

Currently, it is unclear what proportion of aggressive PAs progress into carcinoma. Resistance to medical therapies, however, can be associated with de-differentiation and malignant transformation (31). The mechanism of invasion and metastatic spread is similar to other malignancies, eventually leading to dissemination of tumor cells *via* lymphatic, hematogenous or CSF spread (22).

From an anatomical perspective, it seems likely that anatomical variation plays an important role in the dissemination of tumor cells. The pituitary gland lies in the hypophyseal fossa laterally delineated by the cavernous sinuses. It is unclear whether there is an extra layer between the thin fibrous capsule surrounding the pituitary gland and the cavernous sinuses. Most likely there are variations in thickness, which likely contribute to the variety of invasion in the setting of macroadenomas (32, 33). Similar anatomical variations have been described for the thickness of the diaphragma sellae (34). Thus, at these levels the borders of the hypophyseal fossa are thinner. Additionally, macroadenomas weaken the dura which is supported by the finding that dural invasion increases with tumor size (2).

**Figure 3:**
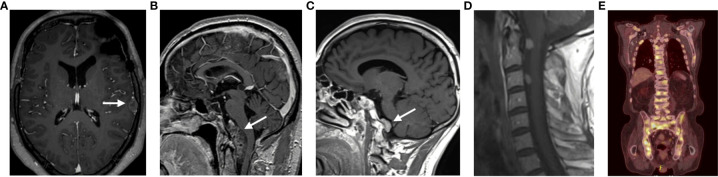
Representation of the different locations of the metastatic lesions of all patients. **(A)** Patient 2; **(B)** Patient 1; **(C)** Patient 3; **(D)** Patient 5; **(E)** Patient 4.

Moreover, during transsphenoidal surgery, these boundaries are easily disrupted and may contribute to the spread of tumor cells in the cerebrospinal fluid (CSF). Similarly, CSF spreading has been reported during transcranial surgery. Taken together, these findings support the hypothesis that surgery could facilitate the development of metastases (35, 36). In our series all cases were diagnosed with a macroadenoma at initial diagnosis, and all had multiple transsphenoidal and/or transcranial resections. Three of the 5 patients developed drop metastases at the craniocervical junction or in the spinal canal. One patient had multiple intraparenchymal lesions and the fourth case developed bone metastases (**Figure 3**).

### Therapeutic options

#### Surgery

In secreting non-prolactinoma macroadenoma and microadenoma unresponsive to medical treatment, transsphenoidal surgical resection is considered the first line treatment. In the presence of important intracranial extension, a transcranial approach may offer advantages. In some cases, both approaches are necessary to obtain a near-total tumor resection. Overall, the endoscopic approach is considered to allow more extensive resections of tumors invading the cavernous sinus and parasellar structures (37). In our series all patients had repeat surgery and 4 out of 5 patients had both surgical approaches (**Table 1**).

#### Radiotherapy

Since the invasive character of PCs, radiotherapy can be used as an adjuvant treatment to obtain a better disease controle.

In general, a radiotherapeutic treatment has a variable long-term tumor control of 80 to 97% and normalizes hormone levels in 40-70% of functioning PAs (38). Both stereotactic radiosurgery (SRS) as fractionated stereotactic radiotherapy (FSRT) are being used. It has been shown that a good disease control can be obtained. In clinical practice, SRS is used for small tumors (<2.5-3cm) in a single-fraction dose of 16 to 25Gy (depending on size and position of the PA), while FSRT is preferred for PAs that are larger and/or nearby the optic tract. The endocrinological outcomes are poor, Hypopituitarisme is the most common complication with an incidence of 30-60% five to ten years after irradiation (39, 40). Far less frequently reported toxicities such as radiation induced optic neuropathy, cerebrovascular accidents and secondary tumors have an incidence of 0-3% (38, 41, 42).

All patients in our series received FSRT after incomplete resection (patient 1 and 5) and/or persistent disease (**Table 1**).

### Medical treatment

#### Standard therapies

In our case series all patients were initially diagnosed with functioning PAs (PRL, PRL/GH and ACTH secreting PAs). As recommended, they were all treated with maximally tolerated doses of antihormonal therapy to control tumor growth (**Table 2**).

In most prolactinomas a reduction of tumor volume and normoprolactinemia can be achieved. Complete resistance only occurs in 10%. Male gender, large tumors and invasive growth are associated with lower response rates (43).

In acromegaly, treatment with somatostatin analogues and the GH receptor antagonist pegvisomant also lead to adequate reduction of tumor volume. Similar to prolactinoma a resistance is seen in 10% of patients (44, 45).

The medical treatment of corticotroph PAs is limited. Therefore, these patients are regularly treated with bilateral adrenalectomy, which often leads to a Nelson’s syndrome. This was also the case in our patients treated with bilateral adrenalectomy. Nelson’s syndrome is also associated with increased tumor growth and progression to metastases (46).

#### Temozolomide

For patients with aggressive PAs resistant to standard therapy, a treatment with the alkylating agent temozolomide (TMZ) is recommended. The first use of TMZ treatment for aggressive PAs was described in 2004. Between 2010 and 2016 eleven studies reported the use of TMZ in the management of PA, refractory to standard treatment. An efficacy of about 37% was reported in a recent ESE survey (47). In all studies, TMZ not only reduced tumor volume but also hormonal levels. Functioning tumors responded better than non-functioning adenomas. MGMT methylation and DNA mismatch repair (MMR) proteins are known predictors of a response to TMZ. A low MGMT and/or an intact MSH6 is associated with a good response to TMZ. Therefore it is recommended have an evaluation of MGMT status by an expert pathologist (15, 47, 48).

Four of the 5 cases were treated with a TMZ treatment (**Table 2**). Two patients had a stable disease, 1 had a partial response and in 2 patients the treatment had to be interrupted due to side effects. In the first case the patient still has a stable disease 4 years after the cessation of TMZ treatment. In our cases the duration of the treatment with TMZ was set at 12 months or until progressive disease, however this remains a point of discussion in literature. In the case series of Santos Pinheiro et al. 82% of patients received TMZ. Moreover all patients treated with a survival that exceeded 5 years were treated with TMZ (29).

Moreover, since TMZ is a known radiosensitizer it is often combined with radiotherapy. An ESE survey indicated that patients treated with concomitant chemoradiotherapy had a better tumor response (47). Additionally, high rates of local tumor control were reported with concomitant chemoradiotherapy as salvage treatment for patients with aggressive PA or PC, especially in patients with MGMT promotor methylation (49). To date the combination of TMZ with other treatments such as capecitabine, pasireotide or octreotide has not been demonstrated (37, 50).

### Alternative treatments for TMZ resistant pituitary tumors

The ESE recommend evaluating the effect after 3 cycles of TMZ. In patients with rapid progression a trial with other systemic cytotoxic or other therapy should be started. In the pre-TMZ era, some antitumoral effect (14%) with limited toxicity was obtained with lomustine/5-fluorouracil.

The use of targeted therapies is emerging in the therapy of PAs. Two cases with therapy-resistant macroprolactinoma were successfully treated with lapatinib, a tyrosine kinase inhibitor against EGFR/HER2. Lapatinib in pituitary tumors is currently being investigated in a phase II clinical trial (NCT00939523) (51). Treatment with other targeted therapies (sunitinib, erlotinib) has not been successful in case reports. An unpublished ESE survey reports good antitumoral responses in a limited number of cases with the anti-VEGF antibody bevacizumab as rescue treatment of in combination with TMZ (15).

#### Immunotherapy

Currently, a variety of malignancies is being treated with great success with immune-checkpoint inhibitors, i.e. inhibition of programmed death 1 (PD-1) and/or cytotoxic T-lymphocyte associated antigen 4 (CTLA-4). Intratumoral expression of PD-L1 and the presence of CD8^+^ tumor infiltrating lymphocytes (TILs) are predictive markers for anti-PD-1 treatment. Recently, investigation of the expression of PD-1 and the presence of CD8^+^TILs in 191 patients with PAs showed that 36.6% had positive PD-L1 expression and 86.9% had CD8^+^ TILs. Functioning PAs had a higher expression of PD-L1(58.8%). Moreover, the PD-L1 expression was significantly associated with higher blood levels of PRL, GH, ACTH and cortisol. PD-L1 expression also correlated with a higher p53 expression (52).

Based on these results and the absence of second line therapy, a treatment with immunotherapy was initiated in the patient 5. Four cycles of ipilimumab (anti-CTLA4 antibody) in combination with nivolumab (antiPD-1 antibody), followed by a maintenance with nivolumab treatment was given with a good clinical result. Unfortunately, there was no new surgical indication, and the PDL-1 expression has not been determined.

A case report recorded a spectacular response to combined treatment with ipilimumab and nivolumab. Furthermore, they performed genomic sequencing on tumors before (pituitary) and after temozolomide treatment (liver) and found a MSH6 mutation in the TMZ-treated liver metastasis (53). MSH6 mutations have been described as a mechanism of tumor resistance to TMZ treatment in glioblastoma (54). Two additional case reports confirmed tumor responses combining ipilimumab and nivolumab (55). Currently, two clinical trials (NCT04042753, NCT02834013) investigate the efficacy and safety of nivolumab and ipilimumab in PAs.

## Conclusion

The diagnosis and treatment of PCs is challenging. Despite the recent update of the WHO classification and the addition of transcription factors, no histological or molecular predictors of aggressiveness have been identified. Moreover, there is a dissociation between the radiological and/or perioperative invasiveness and the immunohistological characteristics of aggressive behavior. This complicates the prediction of the clinical course and decision making towards aggressive treatment. Hence, a multidisciplinary evaluation is fundamental in the follow-up and treatment of these patients.

In this case series we describe 5 cases of PC. Initially 4 of the 5 patients had a good hormonal and radiological tumor control. In the second patient there were more difficulties to obtain tumor control, despite multiple medical and surgical treatments. Since this patient had a high Ki-67 and positive p53 expression, a more aggressive treatment with immediate adjuvant radiotherapy was retrospectively indicated. Although the role of Ki-67 and p53 is controversial, a determination of the Ki-67, mitotic count and p53 at diagnosis should be performed in macroadenoma invading the surrounding structures (on preoperative MRI or perioperative findings). A classification as made by Trouillas et al. combining anatomical and molecular characteristics can be a helpful tool for decision making in these patients.

Corresponding to the literature, treatment with TMZ led to good tumor control in the 3 patients who tolerated the treatment, which supports the treatment of TMZ as first line therapy in PCs. The duration of treatment and indication for concomitant radiotherapy must be further investigated in clinical trials. A rechallenge with TMZ in the fourth patient had no therapeutic effect, which confirms the previously published results. In our 5^th^ patient this led to the initiation of a treatment with check-point inhibition obtaining a stable hormonal and radiological disease for 1 year encouraging the use of immunotherapy in these tumors.

Overall, we can state that further research for predictors of aggressiveness, adequate first and second-line treatments to adequately treat these PCs, is warranted. We recommend the use of the classification of Trouillas for registration and decision making as well as the determination of the transcription factors used in the new WHO classification. Since these are rare tumors, a European registry could capture more insights on the clinical and pathological characteristics and treatments. This data could lead to better diagnosis and individually tailored therapies.

## Data availability statement

The raw data supporting the conclusions of this article will be made available by the authors, without undue reservation.

## Ethics statement

Written informed consent was obtained from the individual(s) for the publication of any potentially identifiable images or data included in this article.

## Author contributions

SDF, JV, DJ and EV reviewed the patient files and documentation and describe the cases. CA and BV overviewed the endocrinological literature, BN the oncological literature and VV and MB the surgical literature. SDF wrote the main text of the manuscript. BV and VV overviewed the manuscript. All authors contributed to the article and approved the submitted version.

## Conflict of interest

The authors declare that the research was conducted in the absence of any commercial or financial relationships that could be construed as a potential conflict of interest.

## Publisher’s note

All claims expressed in this article are solely those of the authors and do not necessarily represent those of their affiliated organizations, or those of the publisher, the editors and the reviewers. Any product that may be evaluated in this article, or claim that may be made by its manufacturer, is not guaranteed or endorsed by the publisher.
